# Seroprevalence of brucellosis and risk factors associated with its seropositivity in cattle, goats and humans in Iganga District, Uganda

**DOI:** 10.11604/pamj.2019.33.99.16960

**Published:** 2019-06-11

**Authors:** Joyce Nguna, Michel Dione, Micheal Apamaku, Samuel Majalija, Denis Rwabita Mugizi, Terence Odoch, Charles Drago Kato, Gabriel Tumwine, John David Kabaasa, Kellie Curtis, Michael Graham, Francis Ejobi, Thomas Graham

**Affiliations:** 1 College of Veterinary Medicine, Animal Resources and Biosecurity, Makerere University PO Box 7062, Kampala, Uganda; 2 Ministry of Health, Uganda; 3 International Livestock Research Institute, Uganda; 4 Veterinarians without Borders, Davis, California, USA; 5 National Agricultural Research Organization, Ministry of Agriculture, Animal Industry and Fisheries, Uganda; 6 University of California Davis, California, USA

**Keywords:** Brucellosis, human, animals, seroprevalence, risk factors, Global Health Security, Uganda

## Abstract

**Introduction:**

The burden of brucellosis among smallholder farmers is poorly-documented in Uganda. The disease burden is likely to be high, given the high levels of endemicity, lots of exposures and due to lack of control measures. In order to designate appropriate control measures, the magnitude and risk factors for brucellosis need to be known. We established the burden of and risk factors for *Brucella* seropositivity in cattle, goats, and humans in Iganga district, eastern Uganda.

**Methods:**

A cross-sectional study was conducted in in Kigulamo Parish, Iganga District. We enrolled 226 households and administered a structured questionnaire to heads of households to capture data on socio-demographic characteristics, human brucellosis-related risk factors, and livestock farming practices. Human, cattle, and goat blood samples were collected and tested serologically using commercial indirect-ELISA kits manufactured by USDA, USA.

**Results:**

Of 451 human blood samples, 20 (4.4%) were positive. Among 345 cattle blood samples, 4 (1.2%) were positive and among 351 goat blood samples, one (0.3%) was positive. Persons who reported consuming locally-made dairy products had 4 times higher odds of *Brucella* seropositivity (OR = 4.0, CI = 1.14-14.03, p = 0.031) than those who did not. None of the risk factors we asked about were significantly associated with seropositivity in cattle and goats.

**Conclusion:**

The seroprevalence of brucellosis in humans in smallholder households in Kigulamo was relatively low and associated with consumption of locally made dairy products. No risk factors were significantly associated with seropositivity in livestock, likely due to the small number of seropositive animals. We recommend a One Health approach to control brucellosis simultaneously in animals and humans needed to sustainably reduce the burden of brucellosis in Uganda and beyond.

## Introduction

Brucellosis is a zoonotic disease caused by *Brucella* species [1], and infects both humans and animals [1, 2]. It exists worldwide, except from countries where eradication of bovine brucellosis has been achieved [3]. The disease is multiple species: those commonly implicated in domestic animals include *Brucella melitensis, Brucella abortus, Brucella canis and Brucella suis*, with their preferential hosts as sheep/goats, cattle, dogs, and pigs, respectively [4]. In humans, the implicated species include *Brucella melitensis* and *Brucella abortus*; thus, goats and cattle are associated with human infection. The prevalence of brucellosis varies widely in animals and humans by country [5]. Globally, approximately 500,000 humans are infected per year [6]. The worldwide economic losses due to brucellosis are extensive not only in animal production but also in human health. In sub-Saharan Africa, the prevalence of brucellosis in humans ranges from 5-55% [6] in different countries, while in domestic animals it is between 8-46% [7]. Infection in humans is attributed to consumption of contaminated animal products such as undercooked meat or unboiled milk and through use of other animal products which are not well-aged or pasteurized [6, 8]. In animals, risk for infections is associated with management factors such as herd size, population density, and herd immunity [8, 9]. Consumption of unboiled milk was significantly (p = 0.004) associated with seropositivity in Mbarara District, however no association was reported among seropositivity with age, sex and awareness of human brucellosis in this study [10]. Mixed livestock or breeds have a higher possibility of infection as opposed to single breed herds [11] and mixing goats and sheep in Eastern and Western Uganda was a probable risk factor [12]. According to Silva et al. extensive grazing, large herd sizes and free grazing are some of the risk factors associated with Brucellosis [13]. Transmission of the disease can also be due to livestock movement from one geographical region with infection to another as well as hygiene factors [1]. In a study carried out by [14] age, entry of purchased animal on the farm, type of breed and sensitization of farmers were outlined as other important risk factors. Brucellosis has been studied previously in different areas of Uganda, where it is known to be endemic. In a study carried out in 2015 in South-western Uganda, the prevalence of brucellosis seropositivity was 14% in cattle, 17% in goats, and 11% in humans [15], and the seroprevalence of brucellosis among exposed cattle-keepers in Mbarara and consumers of unpasteurized milk in Kampala Districts was 5.8% and 9%, respectively [10]. Tumwine *et al.* [16] recorded a seroprevalence of 17% among agro-pastrolists in Kiboga district and seroprevalences of 10% and 7% in Kampala and Mbarara, respectively [17]. The household prevalence of brucellosis in humans from Kiambu county was 5.7% and 31.8% in Kajiado county in neighbouring Kenya and that in animals was 1.2% and 3.4% respectively.

The prevalence of brucellosis is higher in the pastoral grazing areas than in the urban and peri-urban areas [9]. In animals, specifically in cattle population in central and southern Uganda, the individual animal seroprevalence range from 8% to 75.9%, while in goats it was between 4% and 10% by various serological tests [18, 19]. The recent overall individual cattle prevalence documented in Gulu and Soroti was 7.5% and 27.1% overall herd prevalence [20]. A seroprevalence of 12% in bovine samples collected from different laboratories around the country was noted [21]. The prevalence in both humans and animals varies according to geographical location and herd system [22, 23]. In a report by Roushan et al. [24], eastern Iran, a prevalence of 4.0% and 3.9% in humans was recorded using Rose Bengal Plate Test (RBPT) and Enzyme Linked Immunosorbent Assay (ELISA) respectively [24]. Although it has historically been a majority crop-farming area, Iganga district’s livestock industry is steadily growing. This poses the district at risk of zoonotic diseases like brucellosis among its humans and animals. However, there are no data on brucellosis in humans and goats and few data are available on risk factors associated with the spread of brucellosis in livestock as well as humans in Iganga District. Furthermore, there is no program dedicated to the control brucellosis in Uganda. The purpose our study was to estimate prevalence and risk factors associated with *Brucella* positivity in cattle, goats, and humans in Iganga District. This study will also bridge the knowledge gap about prevalence and risk factors of brucellosis among small holder farmers in Uganda.

## Methods

**Study area and design:** this was a cross-sectional study conducted in Makuutu Sub-County, a rural area in Iganga District in the Eastern part of Uganda from June to September 2014. Makuutu Sub-county has 5,203 households with an average of 5 persons in each household and 27,992 people in total [25] and comprises four parishes: Kasozi, Kigulamo, Makandwa and Makuutu. This study was conducted in Makuutu Parish which comprises five villages, including Kigulamo, Bubeto, Naitandu A, Naitandu B and Nakafunvu. Kigulamo Parish was selected for this study because it has a relatively higher number of livestock compared to other parishes. The animal sector in the district is characterized by low productivity with small holder farmers and a total of 125,310 cattle, of which 95.9% are of local breed. Similarly, it has 169,915 goats of which 98.5% are of local breed [25].

**Data collection:** a list of households with at least one herd of cattle or goat in Kigulamo Parish was obtained from local officials and a total of 2 persons per household consented to participate in the study. The sample size for cattle and goats for the study was separately estimated using a sample determination formula described by Thrusfield, 1995 based on 95% confidence level and historical prevalence of 5.5% [9, 26]. Table 1 and Table 2 show the number of humans, cattle and goats sampled in the respective villages in Kigulamo parish. A total of 226 households were selected for the study. A structured questionnaire was administered to the head of the household and this captured data on socio-demographics, biosecurity practices, animal handling, animal product consumption practices, and awareness of brucellosis. For each selected animal, individual animal-level risk factors were captured; sex, age, method of production, history of abortion, breed, herd size, animal management system, vaccination history, and history of introduction of new animals into the herd.

**Table 1:** sero-prevalence of brucellosis among humans by village

Village	No. samples	No. positive	Sero-prevalence (%)
Kigulamo	107	3	0.7
Bubeto	20	2	0.4
Naitandu A	69	4	0.9
Naitandu B	244	11	2.4
Nakafunvu	11	0	0
**Totals**	**451**	**20**	**4.4**

**Table 2:** sero-prevalence of brucellosis in cattle and goats

Individuals	Total tested	Seropositives	Prevalence (%)
All Cattle	345	4	1.2
Male cattle	126	2	0.6
Female cattle	219	2	0.6
All Goats	351	1	0.3
Male goats	66	1	0.3
Female goats	285	0	0

**Laboratory investigation:** in each study household, at least one sample of blood was collected from the respondent who completed the questionnaire and another consenting adult. About 2 ml of venous blood was drawn from each respondent. A total of 451 human blood samples were taken. All cattle and goats in study households were selected. This age cutoff was selected to avoid bias by maternal antibodies creating false positive results. Selected cattle and goats were restrained and 2 ml of venous blood was collected without anticoagulant (Becton DickinsonTM, UK). Serum extraction for both human and animal samples was done at the hematology laboratory at Iganga Hospital and analyzed using ELISA at the Central Diagnostic Laboratory at the College of Veterinary Medicine Animal Resources and Biosecurity (COVAB), Makerere University. Serological testing was done using IgG indirect ELISA following manufacturer’s (USDA, USA) instructions. Two kinds of kits were used; one for humans and the other for cattle and goats, but all were from the same manufacturer.

**Data analysis:** raw data from questionnaires were coded and verified in Microsoft Excel and analysed in SPSS version 17.0 program. All human, cattle and goat suspected risk factors were assessed for their association with brucellosis seropositivity by computing their respective odds ratio and p-values. Variables found to have associations in univariate analysis (p = 0.25) with seropositivity to brucellosis were considered for multivariate modelling.

**Ethics considerations:** ethical approval was obtained from the Mildmay Uganda Research and Ethics Committee (REC REF 0406-2015), registered with the Uganda National Council for Science and Technology (approval Ref #1830) and the Office of the President (ADM 154/212/03). Participation in the study was voluntary with a right of the participant to withdraw at any time without penalty. Informed written consent was obtained and both privacy and confidentiality were ensured. For confidentiality, initials were used for humans and households where samples and data were collected from.

## Results

**Descriptive analysis and laboratory findings:** most respondents were female (61.6%, 278/451) and married (77.9%, 338/451). Muslims comprised the highest percentage (70.3%, 316/451) among religions. Most were farmers (79.4%, 358/451) and 72.1% (325/451) had attained primary school as their highest level of education (Table 3). Of the 451 human samples tested, 20 were sero-positive, giving an overall seroprevalence of 4.4%. The seroprevalence in males was 6.4% (11/173) and in females 3.2% (9/278). The seroprevalence in cattle was 1.2% (4/345) and in goats 0.3% (1/351), while the seroprevalence in male and female cattle was similar (0.6%, 2/351). All female goats tested negative and nearly all (98.8%, 341/345) of the cattle in the study were local breeds. Most households acquired their goats by buying (88.9%, 321/351), and the same applied to cattle (89.9%, 310/345). The most common systems of managing both goats and cattle were a combination of tethering and communal grazing. Regarding breeding, most households used shared males for breeding their goats and cattle. There was no history of abortion in goats and cattle inany households. Most of the goats (94.9%, 333/251) and cattle (76.2%, 363/345) were not vaccinated against brucellosis (Table 4).

**Table 3:** demographic and baseline characteristics of humans

Variable	Characteristic	Frequency (n)	Percentage (%)	P-value
Gender	Male	173	38.4	0.123
	Female	278	61.6	
Occupation	Farmer	358	79.4	0.584
	Formal Employment	60	13.3	
	Non-formal employment	33	7.3	
Education level	No-education	103	22.8	0.505
	Primary	325	72.1	
	Secondary	22	4.9	
	Tertiary	1	0.2	
	Catholic	39	8.6	
Religion	Muslim	316	70.3	0.626
	Anglican	90	20	
	Other Christian	5	1.1	
	Married	338	74.9	
	Single	72	16	
Marital status	Divorced	20	4.4	0.42
	Widowed	21	4.7	

**Table 4:** cattle and goat demographics characteristics

Variable	Distinctives	Goats (%)	Cattle (%)	P-value	
				(Goats)	(Cattle)
Sex	Male	66 (18.8)	126 (36.5)	0.067	0.58
	Female	285 (81.2)	219 (63.5)		
Breed	Local	351 (100)	341 (98.8)	-	0.954
	Exotic	0 (0.0)	1 (0.3)		
	Cross	0 (0.0)	3 (0.9)		
Source	Bought	312 (88.9)	310 (89.9)	0.972	0.835
	Government	5 (1.4)	6 (1.7)		
	Gift	11 (3.1)	4 (1.2)		
Management system	Zero grazing	0 (0.0)	0 (0.0)	0.931	0.853
	Tethering	68 (19.3)	15 (4.3)		
	Fenced farms	0 (0.0)	0 (0.0)		
	Communal	2 (0.6)	17 (4.9)		
	Tethering & communal	281 (80.1)	313 (90.7)		
Breeding method	Shared male	351(100)	344 (99.7)		0.879
	Male not shared	0 (0.0)	1(0.3)		
	Artificial insemination	0 (0.0)	0 (0.0)		
Abortion history	Yes	15 (4.3)	5 (1.4)	0.767	0.732
	No	336 (95.7)	340 (98.6)		
Vaccination history	Yes	18 (5.1)	82 (23.8)	0.745	0.954
	No	333 (94.9)	263 (76.2)		

**Risk factors:** risk factors with p=0.25 in the univariate model (Table 5) were considered for inclusion in multivariable regression and analysed for being indicators of brucellosis (Table 6). In multivariate analysis, only consumption of locally-made milk products (OR = 4.0, CI = 1.14-14.03) was associated with brucellosis seropositivity. Persons who reported consuming locally-made milk products were 4 times more likely to be seropositive than those not consuming. No risk factor was associated with brucellosis in cattle. Only sex and age were associated with *Brucella* seropositivity in goats (Table 7). There were no risk factors found to be significant in multivariabe analysis.

**Table 5:** descriptive statistics and univariable analyses of associated risk factors for seropositivity to Brucella in humans

Variable	Category	N	Positive	P-value
Sex	Male	173	11(6.4)	0.123
	Female	278	9(3.2)	
Knowledge	Yes	191	5(2.6)	0.098
	No	260	15(5.8)	
Have goats	Yes	320	16(5.0)	0.198
	No	125	03(2.4)	
Slaughter from	Home	53	1(2.0)	0.208
	Abattoir	196	18(4.5)	
	Slaughter house	28	0	
Assist at birth	Yes	64	1(1.6)	0.194
	No	381	18(4.7)	
Milk products	Yes	134	10(7.5)	0.037
	No	311	09(2.9)	
Consume milk	Yes	417	19(4.6)	0.112
	No	28	0	

**Table 6:** multivariate logistic regression analysis of risk factors for brucellosis infection in humans

Variable	Category	P value	OR	95% CI	
				Lower limit	Upper limit
Consumption of locally made dairy products	Yes	0.031	4	1.138	14.033
	No	-	1	-	-

**Table 7:** univariable analyses of associated risk factors for seropositivity to Brucella in goats

Variable	Category	N	Prevalence % (ELISA)		P-value
			Positive (%)	Negative (%)
Sex	Male	66	1(1.5)	65	0.067
	Female	285	0	285	
Age	1-8 months	107	1(0.03)	106	0.25
	9-18 months	91	0	91	
	Above 18 months	152	0	152	

## Discussion

The seroprevalence of human brucellosis was 4.4% with no significant statistical difference between males and females. No statistical difference was found in prevalence between males and females (p=0.123, 75% CI) although in this study we found out that more males assisted in animal deliveries and this could probably explain why there was a tendency for males to test more seropositive than females.These findings are lower compared to recent studies in Uganda that recorded a seroprevalence of 5.8% in Mbarara and 9% in Kampala [10], a seroprevalence of 11% in 236 humans carried out in the Southwest [15]. However, in these studies, the study populations were high risk livestock keeping populations with a high dairy industry and with a culture of taking milk as well as locally made milk products. More so, these studies could have been hospital based, with low sample size as compared to Iganga where population was field based. This probably could explain the higher seroprevalence of brucellosis found in Mbarara district compared to Iganga a low risk area with small holder farmers. These results are also consistent with previous studies in humans in Western Iran where the seroprevalence was found to be 3.85% by ELISA [24, 27]. The overall seroprevalence in domestic animals (cattle and goats) was 0.7% (5/696). The seroprevalence of brucellosis was 0.3% (1/351) and 1.2% (4/345) in goats and cattle respectively. These results are in line with those recorded by a study in Nakasongola and Luwero districts where the seroprevalence in cattle was 1.2% and 3.34% on I-ELISA [28]. This low prevalence could be as a result of low numbers of animals kept per household that does not favor disease transmission and spread. Although previous studies implicate communal grazing as a key risk factor for brucellosis in animals, in Iganga where the management system is 100% practiced, the risks remains low possibly because of the very small herd size interrupting transmission. In cattle, no significant difference was observed in infection status between males and females. However, in goats, males tended to be more seropositive compared to the females and this could be associated with their mating behaviour, which exposes them to increased risks of infection. This agrees with previous studies, which found that males were more at risk compared to females [29]. The practice of communal grazing of animals, reported by 88.4% of respondents increases the likelihood of mating with more and this could also partly explain why bucks were likely to be seropositive to *Brucella* antigens than females. These results are much lower than those obtained by previous studies in Mbarara and Eastern Uganda at 4% and 3.4% seroprevalence respectively [30, 31]. However the study results were also divergent from those on cattle and goats where by the higher seroprevalences were obtained in different parts of Uganda [20]. Though the larger difference in the seroprevalences may be attributed to temporal and spatial distributions of brucellosis or variations in the different livestock management systems (larger herd sizes, pasture compared to intensive housing systems). It could also be due to variations in population dynamics, production systems and biological factors. There were no variables found to be significant at multivariate analysis, probably because the number of sero-positives were very small (cattle 4/351, goats 1/345). In cattle, the seropositivity of vaccinated animals (1.25%) was not significantly different (p = 0.95) from those that were not vaccinated (1.16%). Likewise, in goats, no difference was observed in the seroprevalence of *Brucella* seropositivity between vaccinated (0%) and non-vaccinated (0.2%) animals (p = 0.74). This could probably be due to the very low proportion of vaccinated animals or also likely that the vaccines used were not specifically against brucellosis, as respondents were unlikely to distinguish between the different vaccines. The time lapse between vaccination and collection of samples for this study could also affect the outcome of the ELISA tests. We did not find any association between breeds of cattle and goats kept and risk of brucellosis infection in this study. This finding is in line with other studies that recorded no association between the breeds of cattle and brucellosis seropositivity [20]. This was probably because the majority of the animals tested belonged to the same breeds. Data on exotics and cross breeds was scanty and no valid conclusions could therefore be drawn.

On a sub-Saharan scale, the seroprevalence in the current study in cattle and goats are much lower than those of other studies [32] probably because animals in Uganda are more confined compared to other Sub-Saharan countries. At multivariable regression analysis, consumption of locally made milk products significantly increased perceived risk of brucellosis infection. Respondents who consumed the locally made dairy products were believed to be 4 times more likely to test positive to Brucella antigen than those who did not. This agreed with previous studies that revealed that consumption of unboiled milk was significantly associated with brucellosis seropositivity in Mbarara District [10] and Nigeria [33]. No significant statistical difference was found in prevalence between males and females. In this study we found out that more males assisted in animal deliveries and this probably explains why there was a tendency for males to test more seropositive than females.Although no significant differences were found between cattle of age groups below 1 year, 1-2 years and those aged above 3 years, the odds ratio (OR = 2.9) suggested older cattle above 3 years were more likely to test positive for brucellosis than younger cattle. This finding could be due to increased likelihood of exposure with age of cattle. This agrees with previous studies which found age to be a significant risk factor for brucellosis infection in livestock [34, 35]. The tendency to have older animals testing more seropositive than the younger ones disagrees with the findings of Makita [9].

## Conclusion

The seroprevalence of brucellosis in humans in smallholder households in Kigulamo was relatively low. The seroprevalence of brucellosis in cattle and in goats was also low compared to that previously reported in other parts of Uganda. No perceived risk factors were associated with *Brucella* seropositivity in cattle and goats. However consumption of locally made milk products was the major risk factor associated with *Brucella* seropositivity in humans. Nonetheless its impact on public health cannot be underestimated. There is need for a One-health approach to reduce sustainably the burden of brucellosis and other zoonotic diseases in Uganda and beyond.

### What is known about this topic

- Brucellosis is a zoonotic disease affecting both humans and animals characterized by long term febrile illness;
- Prevalence of brucellosis is higher in pastoral grazing areas than in the urban and pri-urban areas; in animals, specifically in cattle population in central and south Western Uganda;
- Infection in humans is attributed to consumption of contaminated animal products and in animals, risk for infections is associated with management factors such as herd immunity.

### What this study adds

- There are no data on brucellosis in humans and livestock and few data are available on risk factors associated with the spread of brucellosis in livestock as well as humans in Iganga;
- Prevalence of brucellosis among small holder farmers is poorly documented in Uganda so this study estimates the prevalence in cattle, goats, and humans in Iganga District;
- This study will also bridge the knowledge gap about prevalence and risk factors of brucellosis among small holder farmers in Uganda.

## Competing interests

The authors declare no competing interests.
